# The Sequential Tissue Distribution of Duck Tembusu Virus in Adult Ducks

**DOI:** 10.1155/2014/703930

**Published:** 2014-08-18

**Authors:** Li Wu, Jinxiong Liu, Pucheng Chen, Yongping Jiang, Leilei Ding, Yuan Lin, Qimeng Li, Xijun He, Qiusheng Chen, Hualan Chen

**Affiliations:** ^1^Key Laboratory of Animal Physiology and Biochemistry, College of Veterinary Medicine, Nanjing Agricultural University, Nanjing 210095, China; ^2^State Key Laboratory of Veterinary Biotechnology, Harbin Veterinary Research Institute, Chinese Academy of Agricultural Sciences, Harbin 150001, China

## Abstract

In 2010, a novel Tembusu virus (TMUV) that caused a severe decrease in the egg production of ducks was isolated in southeast China. Given the novelty of this duck pathogen, little information is available regarding its pathogenesis. Here, we systematically investigated the replication kinetics of TMUV PTD2010 in adult male and female ducks. We found that PTD2010 was detectable in most of the parenchymatous organs as well as the oviduct and intestinal tract from days 1 to 7 after inoculation. Viral titers were maintained at high levels for at least 9 days in the spleen, kidney, bursa of Fabricius, brain, and ovary. No virus was detected in any of these organs or tissues at 18 days after inoculation. PTD2010, thus, causes systemic infections in male and female ducks; its replication kinetics show similar patterns in most organs, with the exception of the ovaries and testes.

## 1. Introduction

A novel Tembusu virus (TMUV), a member of the genus* Flavivirus*, was isolated in a key duck-producing region of southeast China in 2010 [1–3]. Major symptoms of TMUV include a severe decline in egg production in egg-laying and breeder ducks. TMUV was originally detected in mosquitoes captured in Malaysia in 1955 [4] and in the 1970s and 1980s was isolated from mosquitoes of the* Culex* genus in Peninsular Malaysia, East Malaysia (Sarawak), and Thailand. In 2000, a novel TMUV, named Sitiawan virus, was isolated from sick broiler chickens [4]. Generally, birds serve as reservoirs of* Flaviviruses*, although some of these viruses can cause infectious diseases in poultry [5]. Duck TMUV was the first* Flavivirus* determined to cause serious sickness and significant economic loss in the duck industry [1–3].

Duck TMUV has been frequently detected in ducks and other animals in China since the first reported outbreak. This virus has been isolated from mosquitoes in Shandong Province, China [6], and has also been detected in other species including sparrows, geese, and chickens [6–8]. A previous study reported that duck TMUV induced high neurovirulence in intracerebrally inoculated BALB/c mice [9]. Cell-adapted duck TMUV replicates well in most organs of mice [10]. Moreover, TMUV antibodies and RNA have been detected in the serum and from oral swabs obtained from duck farm workers [11]. The rapid spread of this virus and the extensive coexistence of humans and ducks in China highlight the need for attention to be paid to the potential threat to public health caused by the zoonotic nature of* Flaviviruses*.

Because TMUV was only recently identified as a duck pathogen, little information is available regarding its etiological characteristics and pathogenesis. To date, the many reports published on this new pathogen have focused on viral identification [1–3, 12], the establishment of diagnostic methods [13, 14], viral isolation [6, 7, 12], and genetic analysis [15–17]. It has been established that duck TMUV mainly affects adult female ducks (egg-laying and breeder ducks); infection of male ducks remains largely unexplored. Therefore, to clarify the effects of TMUV infection, including its primary replication and tissue distribution in adult male and female ducks, we systematically investigated the viral kinetics and tropism of duck TMUV.

## 2. Materials and Methods

### 2.1. Virus and Cells

TMUV strain PTD2010, isolated from ducks in the Fujian province of China, was propagated for four passages in 9-day-old specific pathogen-free (SPF) embryonated duck eggs. Allantoic fluids were collected and stored at −70°C for further use. Stock titers were detected in duck embryo fibroblasts (DEFs). DEFs were grown in Dulbecco's modified Eagle's medium (DMEM) (Gibco, Carlsbad, CA, USA) supplemented with 10% fetal bovine serum (Hyclone, Logan, UT, USA) plus 100 *μ*g/mL of penicillin/streptomycin (Gibco). All cells were grown at 37°C in an atmosphere of 5% CO_2_.

### 2.2. Animal Studies

A total of 120 8-month-old SPF adult male and female (1 : 1) Shaoxing ducks were provided by the Harbin Veterinary Research Institute, Chinese Academy of Agricultural Sciences (Harbin, China). Of these, 30 male and 30 female ducks were subcutaneously inoculated with a median tissue culture infective dose (TCID_50_) of 10^3^ of PTD2010 in phosphate-buffered saline (PBS). The remaining 30 male and 30 female ducks served as controls. Three ducks from each group were euthanized on days 1, 3, 5, 7, 9, 18, and 35 after infection, and the parenchymatous organs (brain, heart, liver, spleen, lung, kidney, and ovary/testis), digestive tracts (duodenum, jejunum, ileum, caecum, and rectum), and oviducts of the female ducks (infundibulum, magnum, isthmus, shell grand pouch, and vagina) were harvested for viral titration. For histopathological and immunohistochemical analyses of the ovaries and testes, samples were fixed in 10% neutral buffered formalin solution. The remaining ducks were observed daily for signs of sickness until 40 days after inoculation (dpi).

### 2.3. Histopathological and Immunohistochemical Analysis of Ovaries and Testes

After dehydration, tissue blocks of organs were embedded in paraffin and then cut into 4 *μ*m thick sections. Some sections were stained with hematoxylin and eosin and others were used for immunohistochemical analysis, which was performed as described previously [18]. Briefly, after antigen exposure, the sections were incubated with the mouse anti TMUV E protein monoclonal antibody LY12 (prepared by our lab) and subsequently with a biotinylated goat anti-mouse IgG (H+L) secondary antibody (Kirkegaard & Perry Laboratories, Inc., Gaithersburg, MD, USA). After staining, the samples were observed under a microscope (Olympus Corporation, Tokyo, Japan).

### 2.4. Virus Titration

Each tissue sample (0.2 g) was homogenized in 1 mL of PBS, freeze-thawed three times, and centrifuged at 10,000 ×g at 4°C. The supernatant was serially diluted 10^8^-fold from 10^0^ to 10^−8^ with DMEM. DEFs (10^4^) were grown in 96-well plates and infected with 50 *μ*L of the diluted virus cultures. At 72 h after infection, the cells were fixed with 4% paraformaldehyde-PBS for 20 min and then incubated with 0.2% triton X-100 for 15 min to allow permeation. After blocking with 1% bovine serum albumin for 1 h at room temperature, the cells were incubated with the mouse anti TMUV E protein monoclonal antibody LY12 at room temperature for 1 h. After being washed three times with PBS, the cells were incubated for 1 h with Alex Fluor 488-labeled donkey anti-mouse IgG (Life Technologies, Carlsbad, CA, USA). Samples were then observed under an inverted fluorescence microscope (Carl Zeiss Jena GmbH, Jena, Germany). Each sample was tested in triplicate. The TCID_50_ was calculated by using the method of Reed and Muench [19].

## 3. Results and Discussion

**Figure 1:**
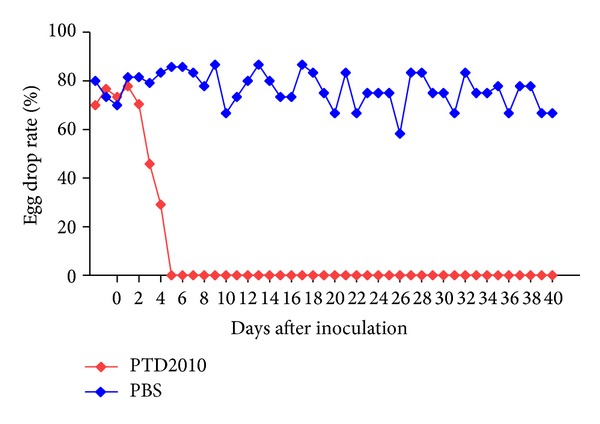
Daily egg production rate after PTD2010 inoculation.

After inoculation with PTD2010, food intake by all infected male and female ducks began to decline at 3 dpi, and green-colored feces was found from 5 dpi. The egg production of infected female ducks declined rapidly from 3 dpi and egg production ceased from 5 dpi (Figure 1). The daily egg production of the control group ducks was 58.3%–86.7%. From 5 dpi, all infected ducks exhibited weight loss, depression, and muddled feathers. Three infected female ducks died at 9, 14, and 15 dpi, respectively, and one infected male duck died at 12 dpi. All surviving, but sick, infected ducks began to recover from 20 dpi; however, no infected female ducks produced eggs during the experimental period.

Necropsies of the infected female ducks clearly showed severe ovarian lesions from 5 dpi, including ovarian hemorrhage, ovaritis, and peritonitis caused by rupturing of the ovarian follicles. Vitellose in the abdominal cavity was completely absorbed by 18 dpi; the ovaries remained in the resting stage and were characterized by small abnormal follicles. Newly formed small ovarian follicles were observed at 35 dpi. However, no obviously identical lesions were found in the testes of the male ducks.

**Figure 2:**
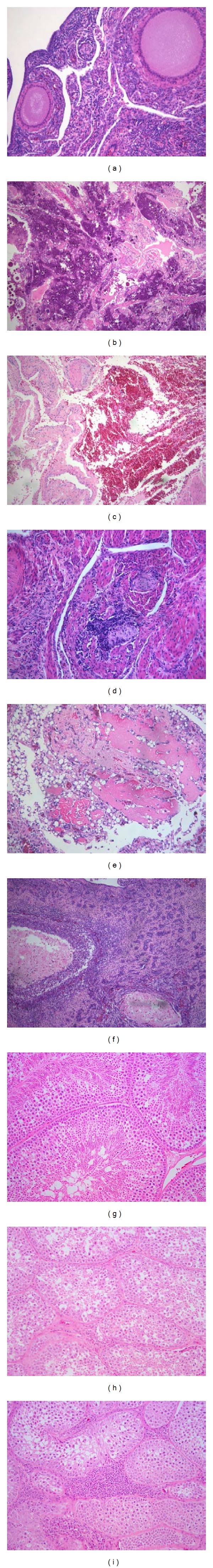
Histopathological analysis of ovaries and testes. Histopathological analysis of the ovary at 5 dpi: mock-infected control (a), ruptured follicles (b), interstitial hemorrhage (c), lymphocytic infiltration (d), smooth muscle degeneration (e), and small vessel hyperplasia (f) can be seen. Pathological changes in the testes at 5 dpi, mock-infected control (g), reduced sperm production, mild interstitial fibrous hyperplasia, spermatocytic swelling, vacuolar degeneration, and desquamation (h) can be seen. The pathological changes progressed, becoming more severe at 9 dpi; focal lymphocytic infiltration was observed (i). Images (a), (d), (e), (g), (h), and (i) were taken at ×200 magnification; Images (b), (c), and (f) were taken at ×100 magnification.

Histopathological analysis of the infected ducks showed that the pathological changes in the ovaries began at 3 dpi and developed to severe changes at 5 dpi (Figures 2(b)–2(f)), characterized by ruptured follicles (Figure 2(b)), interstitial hemorrhage (Figure 2(c)), lymphocyte infiltration (Figure 2(d)), smooth muscle degeneration (Figure 2(e)), and small vessel hyperplasia (Figure 2(f)). Pathological changes in the testes were first observed at 5 dpi, with features that included reduced sperm production, mild interstitial fibrous hyperplasia, spermatocytic swelling, vacuolar degeneration, and desquamation (Figure 2(h)). The pathological changes became more severe at 9 dpi, at which time focal lymphocytic infiltration was noted (Figure 2(i)).

**Figure 3:**
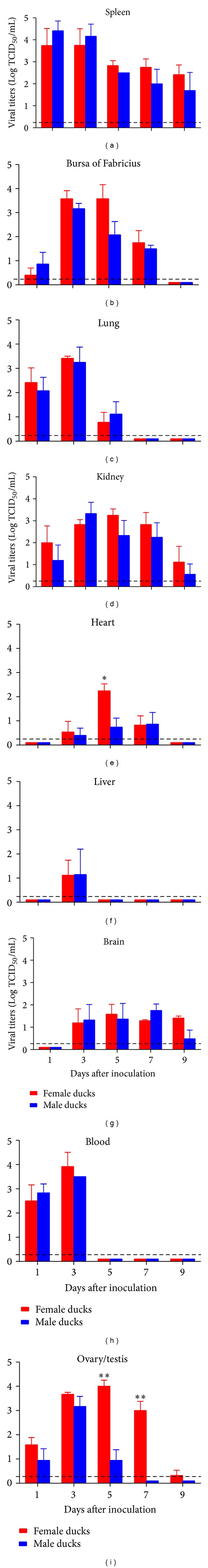
Replication kinetics of PTD2010 in the parenchymatous organs and blood of infected ducks. Data from thespleen (a), bursa of Fabricius (b), lung (c), kidney (d), heart (e), liver (f), brain (g), blood (h), and ovary/testis (i) are shown. Each time point represents the mean viral titer ± SD obtained from three ducks. The black dashed line indicates the limit of detection. *P < 0.05, **P < 0.01; *P* values indicate significant differences in viral titers between male and female ducks.

To investigate the replication of PTD2010 in different parenchymatous organs of male and female ducks, we titrated the tissues collected on days 1, 3, 5, 7, 9, 18, and 35 after inoculation by using DEFs. As shown in Figure 3(a), after inoculation with PTD2010, high viral titers were first detected in the spleen at 1 dpi at 4.42 and 3.74log TCID_50_/mL in male and female infected ducks, respectively. These titers were almost the highest obtained in spleen at all time points after PTD2010 infection and were significantly higher than those in other organs at 1 dpi. From that time point onward, high viral titers in the spleen were maintained and then gradually declined to 1.70 and 2.42log TCID_50_/mL at 9 dpi in male and female ducks, respectively. At 1 dpi, only part of the ducks' bursa of Fabricius exhibited viral replication (Figure 3(b)). At 3 dpi, the viral titers in the bursa of Fabricius reached 3.17 and 3.58log TCID_50_/mL in male and female ducks, respectively, and were maintained at relatively high levels until 5 dpi and then began to decrease by 7 dpi. Viruses were no longer detectable in the bursa of Fabricius of all infected ducks at 9 dpi. Viruses were detected in the lung (Figure 3(c)) and kidney (Figure 3(d)) at 1 dpi, and the mean titers ranged from 2.83log TCID_50_/mL in the kidney to 3.42log TCID_50_/mL in the lung of female ducks at 3 dpi. However, viral titers in the lungs rapidly declined at 5 dpi and disappeared completely at 7 dpi. The viral content in the kidney was maintained at a relatively high level at 5 and 7 dpi, and was detected in one male and two female ducks at 9 dpi. Viral titers in the heart were low and only detectable in one, two, and two of three male ducks and one, three, and two of the three female ducks at 3, 5, and 7 dpi, respectively (Figure 3(e)), whereas the viral titers in the hearts of the female ducks were significantly higher than those in the hearts of the male ducks at 5 dpi (*P* < 0.05). No viruses were detected in liver tissues with the exception of two female and one male duck at 3 dpi (Figure 3(f)). TMUV appeared in brain tissue at 3 dpi, and viral titers were maintained at 1.20–1.58log TCID_50_/mL at 3–7 dpi. At 9 dpi, the brain tissue of one male duck and all three female ducks exhibited viral replication (Figure 3(g)). PTD2010 viremia was transient, being detectable from 1 to 3 dpi (Figure 2(h)), and peak virus titers ranged from 2.75 to 4.5log TCID_50_/mL at 3 dpi. Overall, the increases and decreases in viral titers in all of these organs and in the blood tended were very similar for male and female ducks.

**Figure 4:**
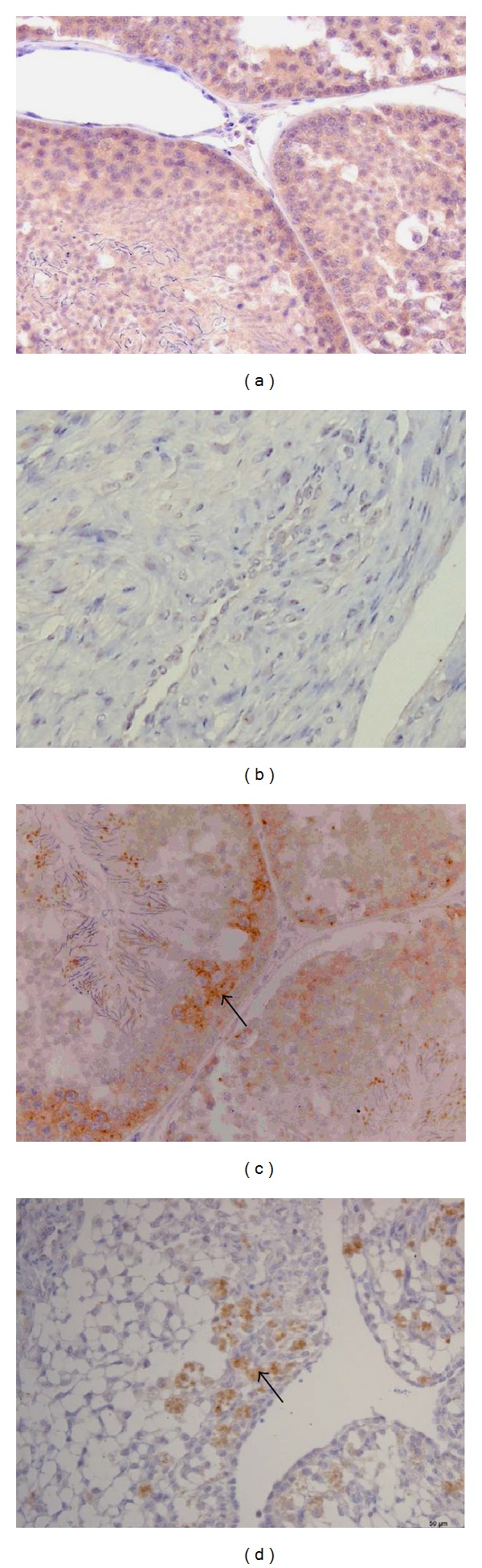
Immunohistochemical analysis of the ovary and testis at 3 dpi. Mock-infected testis (a) and mock-infected ovary (b) are shown; viral antigen was detected in the testis (black arrow) (c) and in the ovary (black arrow) (d). Images (a)–(d) were taken at ×200 magnification.

Significant differences in PTD2010 replication in male and female ducks were detected in the ovaries and testes (Figure 3(i)). Viral titers in the ovaries were maintained at high levels (>3log TCID_50_/mL) from 3 to 7 dpi and were also detectable at 9 dpi. Paradoxically, viral titers in the testes were significantly lower than in the ovaries at 5 dpi (*P* < 0.01), although no viruses were detected in the testes from 7 dpi. The immunohistochemical results revealed viral antigens in the ovaries and testes at 3 dpi (Figure 4).

**Figure 5:**
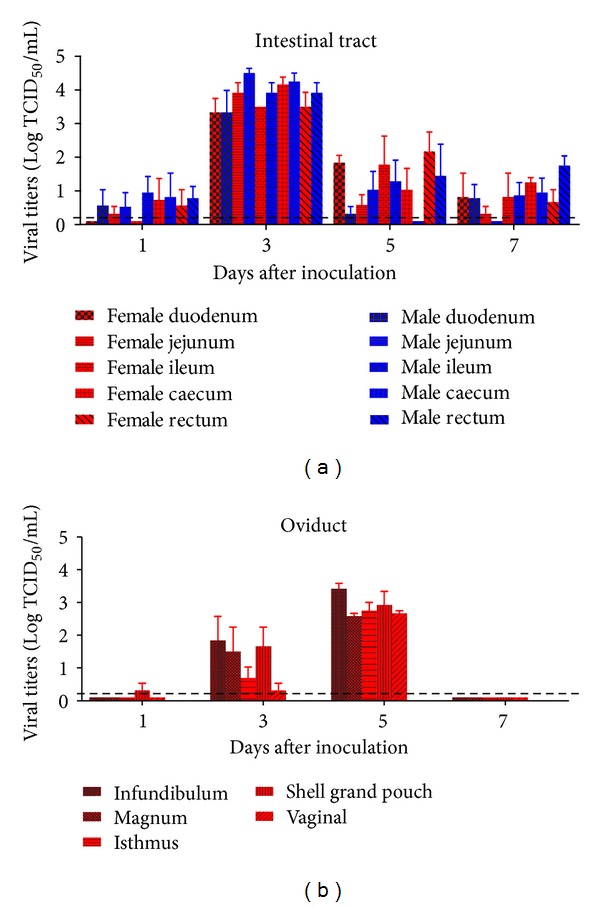
Replication kinetics of PTD2010 in the intestinal tract and oviduct of infected ducks. Data from the digestive tract (a) and oviduct (b) are shown. Each time point represents the mean viral titer ± SD obtained from three ducks. The black dashed lines in (a) and (b) indicate the limit of detection.

In this study, we also investigated the replication of PTD2010 in the intestinal tracts of male and female ducks. As shown in Figure 5(a), PTD2010 replication was detected in five segments of the intestinal tracts, namely, the duodenum, jejunum, ileum, caecum, and rectum, of both male and female ducks at 1 dpi. Subsequently, the viral titers in the intestinal tracts rapidly increased to peak titers of 3.3–4.5log TCID_50_/mL in male ducks and 3.3–4.17log TCID_50_/mL in female ducks at 3 dpi. From that time point onward, the viral titers gradually decreased at 5 and 7 dpi and disappeared completely at 9 dpi, as was observed in some of the parenchymatous organs. No obvious differences were observed between the male and female ducks; however, PTD2010 replication in the oviducts of the female ducks was obviously delayed compared with that in the intestinal tracts. As shown in Figure 5(b), we looked for PTD2010 in five portions of the oviduct: the infundibulum, magnum, isthmus, shell grand pouch, and vagina. Viruses were first detected in the oviducts of some ducks at 3 dpi. These viral titers significantly increased from 3 to 5 dpi, and viruses were detected in every part of the oviduct of all female ducks by 5 dpi. However, the viruses were rapidly eliminated from the oviduct at 7 dpi. The peak mean titers in the oviduct, ranging from 2.58 (infundibulum) to 3.42log TCID_50_/mL (magnum), were lower than those in the intestinal tracts at 5 dpi. Moreover, we found that the viral titers were not obviously different among the different sections of the intestinal tracts and oviducts.

The* Flavivirus* infection in animal hosts usually goes through three phases, including initial infection and spread, peripheral viral amplification and neuroinvasion [20]. However, in this study, high titers of TMUV were detected in the blood, spleen, lung, and kidneys of ducks as early as 1 dpi and in the brains of ducks as early as 3 dpi. The rapid replication, spread, and neuroinvasion of TMUV in ducks may due to the high inoculated viral titers in the tests.

## 4. Conclusions

In summary, PTD2010 caused systemic infections in female and male ducks and was detected in most parenchymatous organs as well as the oviducts and intestinal tracts from 1 to 7 dpi. Viruses were detectable in only four organs of female and three organs of male ducks at 9 dpi. No virus was detected in any of the examined organs or tissues after 18 dpi. The viruses showed similar replication kinetics in female and male ducks, except in the ovary and testis, where the replication kinetics differed only slightly. Although the viral titer in the testes was significantly lower than that in the ovaries at 5 dpi, the pathological effects were very severe, causing total destruction of sperm generation. This study is the first to systematically assess the viral load and tissue distribution of TMUV in both male and female adult ducks. Our results provide further insights into the pathogenesis of TUMV in ducks.
